# The Changes That Occur in the Immune System During Immune Activation in Patients With Prediabetes From All Ethnicities, Aged 25-45 Years: Protocol for a Systematic Review and Meta-analysis

**DOI:** 10.2196/31619

**Published:** 2022-11-14

**Authors:** Nomusa Christina Mzimela, Aubrey Mbulelo Sosibo, Phikelelani Siphosethu Ngubane, Andile Khathi

**Affiliations:** 1 Department of Human Physiology College of Health Sciences, School of Laboratory Medicine and Medical Science University of KwaZulu Natal Durban South Africa

**Keywords:** systematic review, meta-analysis, prediabetes, immune cells, inflammatory markers, diabetes, inflammatory response, immunology, demographics, risk factors

## Abstract

**Background:**

Prediabetes is an asymptomatic, intermediate state between normoglycemia and the onset of type 2 diabetes mellitus. Recent reports indicate that during prediabetes, there are subclinical changes to immune cells and inflammatory markers. Therefore, this systematic review will provide a synthesis of the available data on the changes in the concentration of immune cells and selective inflammatory markers. It will also give evidence of a demographic impact on changes or complications in the prediabetes state.

**Objective:**

The objectives of this study are to create a protocol that will be used to analyze the collected data of previously published research based on immune cells such as neutrophils, lymphocytes, monocytes, eosinophils, and basophils, as well as inflammatory markers such as C-reactive protein, tumor necrosis factor-alpha, interleukin-6, P-selectin, cluster of differentiation 40 ligand, and fibrinogen. Additionally, an impact of demographics will be determined using the previously published data collected.

**Methods:**

This protocol was prepared through adhering to the PRISMA (Preferred Reporting Items for Systemic Reviews and Meta-Analysis) 2015 guidelines for reporting protocols. Published clinical studies that involve observational (cross-sectional, comparative cross-sectional, case-control, or cohort) study designs that include normal or nondiabetic and prediabetes reports will be used in this systematic review and meta-analysis. This will be accomplished by using clinical Medical Subject Headings to search on MEDLINE, Cochrane library, and African Journal Online. Reviewers (NCM, AMS, and AK) will screen all the results and select the studies that meet the eligibility criteria. Downs and Black Checklist will be used to check the risk of bias, and then a Review Manager v5.4 forest plot will be used for meta-analysis. Additionally, the forest plot will also be used for sensitivity analysis. The strength of evidence will then be assessed using the Grading of Recommendations Assessment, Development, and Evaluation (GRADE) approach.

**Results:**

Since July 5, 2020, there are no participants recruited. Publicly available data will be used in the review and will be collected after this protocol publication. No ethics approval is required as no subjects will be used, and analysis will be based on reported data. Authors will be contacted if there was a misunderstanding related to reading their reported data.

**Conclusions:**

The findings will clarify changes that might be observed in a study of interest based in the eThekwini district in South Africa.

**Trial Registration:**

International Prospective Registry of Systematic Reviews (PROSPERO) CRD42020184828; https://www.crd.york.ac.uk/prospero/display_record.php?RecordID=184828

**International Registered Report Identifier (IRRID):**

PRR1-10.2196/31619

## Introduction

Type 2 diabetes (T2D) is a metabolic disorder characterized by chronic hyperglycemia, which gives rise to metabolic and signaling abnormalities [1-3]. According to Kayal and Graves [3], these metabolic and signaling abnormalities have been reported to cause dysregulated innate immunity. Chronic dysregulated immunity includes changes in immune cells such as neutrophils, lymphocytes, monocytes, eosinophils, and basophils [4-7]. Upon activation, these immune cells play a different role, including secretion of inflammatory markers such as C-reactive protein (CRP), tumor necrosis factor-alpha (TNF-α), interleukin-6 (IL-6), P-selectin, cluster of differentiation 40 ligand (CD40L), and fibrinogen [4,5,8-11]. The chronic immune activation in T2D results in a suppressed immune system [3,12]. According to Lam and LeRoith [13], a fundamental change in the population with T2D is witnessed by the health care communities. This was confirmed by the International Diabetes Federation statistics, reporting that in 2019, there were 19 million people with diabetes in Africa aged 20-79 years [1]. The International Diabetes Federation also reported that there were 12 million Africans aged 20-79 years living with undiagnosed diabetes in 2019 [1]. South Africa is the highest with 4.6 million adults with diabetes (20-79 years) [1]. In 2017, the Indian population was reported to have the highest prevalence of diabetes in South Africa by 11%-13%, followed by people of color by 8%-10%, then Black people by 5%-8%, and White being the lowest by 4% [14,15]. The Indian population among people with diabetes has been shown to be high due to their strong diabetes genetic predisposition [14,15]. However, the onset of T2D arises from the progression of prediabetes [16]. Prediabetes has been reported to be an asymptomatic state, creating a research complication in its documentation of the statistics and prevalence. There is less evidence on the changes in immune cells and selective inflammatory markers at the prediabetes stage [17-20]. However, in our laboratory, research has been conducted on animals in addition to the available research reporting the metabolic and signaling abnormalities, including immune activation during prediabetes [21-24]. This then raised a debatable issue if the same abnormalities occur during prediabetes in human individuals owing to limitations in the animal models, even though the research mimicked the human diet. From the search conducted, we found no report or evidence of the systematic review that reports on the changes in immune cell concentration and the level of secretion of selective inflammatory markers that occur during immune activation during prediabetes. Therefore, our research presents an opportunity to compile a systematic review that will yield an exhaustive synthesis obtained from the available studies that previously reported on immune cells and selective inflammatory markers of interest in prediabetes. Additionally, this systematic review will give reports on the impact of demographics on changes of immune cells and secretion of selective inflammatory markers during prediabetes.

The objectives of this study are as follows: (1) to determine the changes in concentration of immune cells, such as neutrophils, lymphocytes, monocytes, eosinophils, and basophils during prediabetes; (2) to investigate if there are changes in concentration on selected inflammatory markers, such as CRP, TNF-α, IL-6, P-selectin, CD40L, and fibrinogen, during prediabetes; and (3) to assess the variation of prediabetes-associated changes in immune function among different demographic groups.

## Methods

This protocol was prepared by adhering to the PRISMA (Preferred Reporting Items for Systemic Reviews and Meta-Analysis) 2015 guidelines for reporting protocols [25].

### Systematic Review Registration

The protocol has been registered with PROSPERO with registration number “CRD42020184828,” dated July 5, 2020.

### Ethics Approval and Consent to Participate

The data analyzed will be those that have already been published, and there will be no data collection from individuals. The authors declare that there will be no informed consent required to be signed; therefore, no ethics approval is required for the systematic review and meta-analysis.

### Eligibility Criteria for the Study

Studies with a minimum of 100 participants (N=100) and the studies that report community-based clinical cross-sectional study will be eligible. The inclusion and exclusion criteria will be as follows: inclusion—the information reported from nondiabetic adult patients aged 25-45 years from all ethnicities will be used; exclusion—information reported from people with a history of liver disease, kidney disease, heart disease, and depression will not be used. Information from pregnant women will also not be used. Additionally, no samples from professional sports athletes will be allowed in the study. Full-text articles or reports indicating that individuals who were used were free from all the mentioned criteria will then be eligible.

### Prediabetes Diagnosis Criteria

Diagnostic criteria for prediabetes will be as follows (participants should meet 1 of the following diagnoses): fasting blood glucose—5.6 to 7.0 mmol/L; 2 hours postprandial blood glucose (2 hours oral glucose tolerance test)—7.8 to 11.0 mmol/L with glycated hemoglobin (5.7%‐6.4%).

### Study Design

#### Participants

##### Intervention

These will be clinical studies that involve observational studies if they are cross-sectional, comparative cross-sectional, case-control, or cohort study designs that involve normal (nondiabetic) and prediabetes reports. The reported information that involves one or more immune cells (neutrophils, lymphocytes, monocytes, eosinophils, and basophils) in the prediabetic state will be eligible for this systematic review. Additionally, studies that report information that involves at least one or more inflammatory markers of interest, which are CRP, TNF-α, IL-6, P-selectin, CD40L, and fibrinogen, will also be eligible for this systematic review.

##### Comparators

In this systematic review, the eligible comparing control groups will be normal (nondiabetic) control and T2D control groups.

### Outcomes

This systematic review is expected to show the following results: (1) the changes in concentration of immune cells such as neutrophils, lymphocytes, monocytes, eosinophils, and basophils during prediabetes (reported as odds ratio and 95% CI); (2) the changes in concentration of selected markers such as CRP, TNF-α, IL-6, P-selectin, CD40L, and fibrinogen during prediabetes (reported as odds ratio and 95% CI); and (3) variations in markers of immune function among different demographic groups based on gender, age, and race (reported as the mean).

### Search Strategy

The electronic search strategy will be used as an identification for studies involving cohorts that have been studied that are related to the study of interest [26]. This strategy will be accomplished by search on MEDLINE (from 1963 to 2020), Cochrane library displaying results of trials from PubMed, CT.gov, Embase, ICTRP (from 1963 to 2020), as well as African Journal Online (from 1998 to 2020) [26]. In addition to these search strategies, clinical MeSH (Medical Subject Headings) and text will be applied to filter the available information. For all search conducted, the keywords to be used will be “pre-diabetes and immunity,” “pre-diabetes and immune cells,” “pre-diabetes and leucocytes,” and “pre-diabetes and inflammation.”

### Identification of Eligible Studies

The title and abstracts of all the obtained results will be screened by reviewers (NCM, AMS, and AK), and the studies that meet the eligibility criteria will then be selected. Each reviewer will be responsible for screening all the selected study reports before the decision-making of the eligible reports. The PRISMA flowchart for selection of studies will then be provided in the reports from the systematic review.

### Patient and Public Involvement

No patient was involved.

### Data Management

#### Study Records and Data Extraction

The data of the study records that are selected as eligible reports will then be extracted and recorded in an Excel (Microsoft Corp) file. The predefined list of variables to be considered in each report will be used as categories in an Excel file. Considering the research of interest, the outcome of interest will mainly be the immune cell response and concentration of selected markers in both genders, at an age parameter of interest, and in all ethnicities. However, the value of the baseline characteristic of the data reported will also be considered. Therefore, the baseline characteristics of the eligible research reports obtained will be author, year of publication, country, and study setting. The methodology of the study reported will also be considered with categories including design, period, sampling strategy, and whether participants are from a normal or prediabetic population. Finally, the outcomes from different genders, ages, ethnicities, and immune cell changes or inflammatory markers will then be extracted.

#### Data Simplification

Studies that report on the immune cells (neutrophils, lymphocytes, monocytes, eosinophils, and basophils) will be combined into a single group. Additionally, the studies that report on selected inflammatory markers (CRP, TNF-α, IL-6, P-selectin, CD40L, and fibrinogen) will also be combined into a single group.

#### Risk of Bias

The potential risk of bias in individual studies will be obtained using the Downs and Black Checklist [27]. The scores will be rated as follows: excellent (25-26), good (20-24), moderate (14-19), poor (11-13), and very poor (<10). Three reviewers (NCM, AMS, and AK) will be responsible for the independent judgments, which will be based on the 4 domains of the Black and Downs checklist tool, which are reporting bias (10 items), external validity (3 items), internal validity (6 items), and selection bias (7 items). In a situation where there will be a difference of opinions between NCM, AMS, and AK, author PSN will be responsible for adjudication. In situations where the data are not clear, the investigator who reported the data will be contacted 3 times. If no response is obtained, data will be then excluded from the eligible report.

#### Data Synthesis

For the meta-analysis of reported data, a forest plot will be used from Review Manager software version 5.4 (RevMan) [28-30]. Using this forest plot, eligible data from all reported studies will be analyzed depending on their sample size and the mean of the concentration of immune cells or inflammatory markers in prediabetic and control groups. Additionally, an odds ratio and CI will be used to make the forest plot where the solid lines will represent the 95% CI. Each reported study will be represented as a horizontal line on the y-axis to list the primary author and year of study. The forest plot will also include the weight of the study results that will be automatically obtained using the Review Manager software.

### Sensitivity Analysis

RevMan forest plot will also test for heterogeneity, where greater homogeneity will be indicated by a greater overlap between the CIs [30]. Using the forest plot, *I*^2^ will then be calculated where a value between 0% and 100% will be obtained. A value obtained less than 25% will be an indication of a strong homogeneity, and a value obtained greater than 75% will be an indication of a strong heterogeneity. However, a value of 50% will be considered as an average value.

### Assessment of Strength of Evidence

NCM, AMS, and AK will then be responsible for the assessment of the strength of evidence. The studies included in the review will then be evaluated using the Grading of Recommendations Assessment, Development, and Evaluation approach (GRADE) [30-32]. Furthermore, the summary of findings table will then be created using a GRADEpro (McMaster University and Evidence Prime Inc) tool.

## Results

As of July 5, 2020, no participants have been recruited as publicly available data will be used. These data will be collected when this protocol has been published. There will be no ethics approval required as the review is based on published data, and authors will be contacted if there is a misunderstanding from reading their reported data for clarity of their published work.

## Discussion

### Principal Findings

The synthesis of previous study reports obtained from this systematic review and meta-analysis will clarify the complications on the immune system at prediabetes such as the changes that have been reported on immune cells, which are neutrophils, lymphocytes, monocytes, eosinophils, and basophils. This systematic review and meta-analysis will also give an outstanding synthesis of data from previous reports based on selected inflammatory markers of interest.

### Conclusion

The synthesis from this systematic review and meta-analysis will create a hallmark of association between demographics and prediabetes. This will clarify changes that might be observed in a study of interest based in the eThekwini district in South Africa.

## Abbreviations

CD40L: cluster of differentiation 40 ligands
CRP: C-reactive protein
GRADE: Grading of Recommendations Assessment, Development, and Evaluation
IL-6: interleukin-6
MeSH: Medical Subject Headings
PRISMA: Preferred Reporting Items for Systemic Reviews and Meta-AnalysisT2d: type 2 diabetes
TNF-α: tumor necrosis factor-alpha
